# Extracellular CIRP Induces Macrophage Extracellular Trap Formation *Via* Gasdermin D Activation

**DOI:** 10.3389/fimmu.2021.780210

**Published:** 2021-12-23

**Authors:** Yongchan Lee, Bridgette Reilly, Chuyi Tan, Ping Wang, Monowar Aziz

**Affiliations:** ^1^ Center for Immunology and Inflammation, The Feinstein Institutes for Medical Research, Manhasset, NY, United States; ^2^ Department of Surgery, Zucker School of Medicine at Hofstra/Northwell, Manhasset, NY, United States; ^3^ Department of Molecular Medicine, Zucker School of Medicine at Hofstra/Northwell, Manhasset, NY, United States

**Keywords:** eCIRP, macrophages, extracellular traps, METs, gasdermin D, caspase-1, MPO

## Abstract

Extracellular cold-inducible RNA-binding protein (eCIRP) is a damage-associated molecular pattern promoting inflammation and tissue injury. During bacterial or viral infection, macrophages release DNA decorated with nuclear and cytoplasmic proteins known as macrophage extracellular traps (METs). Gasdermin D (GSDMD) is a pore-forming protein that has been involved in extracellular trap formation in neutrophils. We hypothesized that eCIRP induces MET formation by activating GSDMD. Human monocytic cell line THP-1 cells were differentiated with phorbol 12-myristate 13-acetate (PMA) and treated with recombinant murine (rm) CIRP. The MET formation was detected by three methods: time-lapse fluorescence microscopy (video imaging), colorimetry, and ELISA. Cleaved forms of GSDMD, and caspase-1 were detected by Western blotting. Treatment of THP-1 cells with rmCIRP increased MET formation as revealed by SYTOX Orange Staining assay in a time- and dose-dependent manner. METs formed by rmCIRP stimulation were further confirmed by extracellular DNA, citrullinated histone H3, and myeloperoxidase. Treatment of THP-1 cells with rmCIRP significantly increased the cleaved forms of caspase-1 and GSDMD compared to PBS-treated cells. Treatment of macrophages with caspase-1, and GSDMD inhibitors z-VAD-fmk, and disulfiram, separately, significantly decreased rmCIRP-induced MET formation. We also confirmed rmCIRP-induced MET formation using primary cells murine peritoneal macrophages. These data clearly show that eCIRP serves as a novel inducer of MET formation through the activation of GSDMD and caspase-1.

## Introduction

Cold-inducible RNA-binding protein (CIRP) is an 18-kDa nuclear protein (1). During sepsis, trauma, hemorrhage, and organ ischemia–reperfusion injury, nuclear CIRP is translocated to the cytoplasm and released outside the cells to serve as a damage-associated molecular pattern (DAMP), worsening inflammation and tissue injury (1, 2). Macrophages treated with lipopolysaccharide (LPS) or exposed to hypoxia causes the release of CIRP extracellularly through lysosomal exocytosis or necrosis (1). Extracellular CIRP (eCIRP) activates macrophages, neutrophils, and lymphocytes by binding to Toll-like receptor 4 (TLR4) and/or triggering receptor expressed on myeloid cells-1 (TREM-1) (2, 3).

While the innate immune cells, *i.e.*, neutrophils and macrophages, serve as the first line of defense against the pathogen, their exaggerated activation and effector functions lead to severe inflammation and tissue injury in sepsis (4–6). Upon bacterial and viral infection or following stimulation of neutrophils with eCIRP and cytokines/chemokines, they produce neutrophil extracellular traps (NETs), which contain chromatin decorated with citrullinated histone H3 (citH3), myeloperoxidase (MPO), and anti-microbial peptides (7, 8). eCIRP induces NET formation by activating peptidyl arginine deiminase 4 (PAD4), which causes citrullination of histone H3 (9). In addition, recent reports indicate that the pore-forming protein gasdermin D (GSDMD) that causes pyroptosis, a form of inflammatory cell death, can promote NET formation (10, 11). During NETosis, GSDMD can be directly activated by neutrophil proteases or indirectly by the noncanonical inflammasome involving caspase-11 activation. As such, a small molecule that inhibits GSDMD-mediated pyroptotic cell death can inhibit NET formation (11). Akin to neutrophils, macrophages have been shown to form macrophage extracellular traps (METs) to entrap and kill various microbes (12–14). An analysis of the structure of METs revealed that they are composed of nuclear and mitochondrial DNA, and also MPO and lysozyme-proteins previously identified in NETs (14–16). Studies have demonstrated the generation of METs by several macrophage subsets and cell lines in response to a wide range of microbes and their products (13, 14). While ample evidence shows NET production in response to DAMPs (7), their effects on METosis remain unexplored. MET release has been mainly focused on murine primary macrophages or macrophage cell lines (14). However, in human macrophages, the release of METs and their formation mechanism are not thoroughly studied.

Previously, eCIRP has been shown to stimulate the NLRP3 inflammasome pathway, activating caspase-1 and causing pyroptotic cell death in lung endothelial cells (17). GSDMD is cleaved by activated caspase-1 downstream of canonical inflammasome activation or caspase-4, -5, and -11 upon their ligation and activation by cytosolic LPS (10, 18). In this study, we hypothesized that eCIRP induces the GSDMD pathway in macrophages to promote MET formation. Our results demonstrated that stimulation of human macrophages with eCIRP induces caspase-1-dependent GSDMD activation that triggers METosis. Identification of eCIRP’s new role in macrophages will provide valuable insights into the pathophysiology of inflammatory diseases in which uncontrolled activation of innate immune cells is a significant problem.

## Materials and Methods

### Reagents

Recombinant murine CIRP (rmCIRP) was prepared in-house as described previously (1). Disulfiram was purchased from Sigma-Aldrich (cat. no. D2950000, Saint-Louis, MO), Pan-caspase inhibitor z-VAD-fmk from Selleckchem (cat. no. S7023), deoxyribonuclease I (DNase I, cat. no. 18047-019), Hoechst 33342 (cat. no. R37605), SYTOX Green (cat. no. S7020), SYTOX Orange (cat. no. S11368), and SYTO Deep Red DNA stain (cat. no. S34900) from Thermo Fisher Scientific (Waltham, MA). Antibodies against full-length gasdermin D (cat. no. 39754), cleaved gasdermin D (cat. no. 36425), and cleaved caspase-1 (cat. no. 4199) were purchased from Cell Signaling Technology (Danvers, Massachusetts), antibody against full-length caspase-1 (cat. no. AG-20B-0048-C100) from Adipogen Life Sciences (San Diego, CA), antibody against human IL-1β that detects both pro- (full-length) and mature (cleaved) IL-1β (cat. no. 16806-1-AP) from Proteintech (San Diego, CA) (19), antibody against β-actin (cat. no. A5441) from Sigma, anti-MPO-FITC (ab90812), and anti-histone H3 (citrulline R2 + R8 + R17) antibody (ab5103) from Abcam (Waltham, MA). Anti-MPO-biotin antibody (HM1051BT) was purchased from Hycult Biotech (Uden, The Netherlands). Anti-TLR4 antibody (cat. no.: 312813) and IgG isotype control Abs (cat. no. 400263) were purchased from Biolegend (San Diego, CA). Human IL-1β ELISA kit (cat. no.: 88-7261-22) was purchased from Invitrogen (Waltham, MA).

### Animals

Male, C57BL/6 mice (8–12 weeks old) were purchased from Charles River Laboratories (Kingston, NY). Mice were housed in temperature-controlled environments and fed a standard laboratory mouse chow and drinking water. We boarded the mice in a room, maintaining a 12-h light/dark cycle. All animal experiments were performed following the National Institutes of Health guidelines for the care and use of laboratory animals. This study was approved by the Institutional Animal Care and Use Committee of the Feinstein Institutes for Medical Research.

### Cell Culture

Human monocytic cell line THP-1 cells were obtained from ATCC (TIB-202) and maintained in RPMI media with fetal bovine serum (10%), penicillin–streptomycin, and 2-mercaptoethanol (50 µM). The cells were differentiated with 90 ng/ml phorbol 12-myristate-13-acetate (PMA) for 24 h, followed by resting for 24 h in regular media without PMA. The cells were then replenished with fresh media immediately before any treatment was used in this study. Mouse peritoneal macrophages isolated by lavage were cultured in RPMI media with fetal bovine serum (10%), penicillin–streptomycin. All cells were maintained at 37°C, 5% CO_2_ in humidified tissue culture incubator. Cell culture samples were collected at 3, 6, and 16 h after treatment of rmCIRP unless otherwise indicated. The treatment of inhibitors was preceded 30 min earlier than the treatment of rmCIRP. The incubation of cell staining dye was done 30 min before the treatment of inhibitors.

### METs Assay by a DNA NanoDrop Spectrophotometer

The extracellular DNA released from THP-1 cells treated with rmCIRP was measured by UV spectrophotometer as described previously (20, 21). THP-1 cells were cultured in 6-well culture plate and treated with rmCIRP at 1 µg/ml for the desired time point. The plate was centrifuged for 10 min at 200×*g*. The culture media was removed. PBS was added to the well and vigorously pipetted. The METs and the cells were scraped using a cell scraper. The cell suspension was transferred to a microcentrifuge tube and centrifuged for 10 min at 300×*g*. The cell pellet was discarded, and the supernatant was collected and centrifuged for 15 min at 13,000×*g*. The pellet was suspended in PBS. The concentration of DNA in the suspension was measured by a NanoDrop spectrophotometer (ThermoFisher Scientific).

### MPO–DNA ELISA

The MPO–DNA was measured by ELISA as described previously (20, 21). Streptavidin-coated strip well plate (Thermo Fisher Scientific) was coated with anti-MPO-biotin in sodium bicarbonate buffer (pH 9.5) overnight at 4°C and blocked with 1% BSA in PBS for 2 h at room temperature, followed by the incubation of extracellular DNA from THP-1 cells treated with rmCIRP. The DNA samples were diluted 4 times and incubated for 2 h while agitating vigorously (300 rpm on a rotary shaker) and then incubated overnight at 4°C. The extracellular DNA prepared from the cells treated with rmCIRP (5 µg/ml) was used to make a standard curve by 1:2 serial dilution and incubated along the samples prepared. The plate was washed with PBS with Tween 20 (PBST) 3 times and incubated with anti-DNA-antibody–peroxidase conjugate (Roche cell death ELISA kit, cat no.: 11544675001) for 2 h at room temperature with gentle agitation in a rotary shaker. The plate was washed with PBST for 5 times. TMB substrate was added to the plate and incubated for 15 min, and the reaction was stopped by adding a stop solution (2N sulfuric acid). Biotek Synergy H1 plate reader (Winooski, VT) measured the optical density.

### Western Blotting

The lysate of THP-1 cells was prepared by adding 100 µl RIPA buffer to each well of a 12-well plate. The RIPA buffer was supplemented with 2 mM Sodium Orthovanadates, 0.2 mM phenylmethylsulfonyl fluoride (PMSF), and cOmplet mini-protease inhibitor cocktail from Roche (cat. No.: 11836153001; Millipore, Sigma, St. Louis, MO). The protein concentration of the lysate was determined with a protein assay reagent (cat. no.: 5000002, Bio-Rad, Hercules, CA). Protein samples (20 µg each) were separated by SDS-PAGE using 4–12% Bis-Tris gel (Invitrogen, NP0322, ThermoFisher Scientific) and transferred to nitrocellulose membrane by X cell II blot module. The membrane was incubated with primary antibody overnight at 4°C, followed by the incubation of Odyssey secondary antibody (cat. no.: 926-32211 or 926-68070, Lycor Biosciences, Lincoln, Nebraska). The detection of Western blot was done by Odyssey CLx imaging system (Lycor Biosciences).

### Time-Lapse Microscopy

Time-lapse microscopy was performed with a Nikon Eclipse *Ti* microscope equipped with a motorized stage, a DS-Qi2 monochrome camera, perfect focus system, and SOLA 6-LCR-SC lightbox (Lumencor light engine). For live-cell image, EVOS onstage incubator system (Thermo Fisher Scientific) was mounted on the stage for maintaining temperature (37°C), humidity (80% or higher), and gas supply (5% CO_2_). Nikon Element advance research software was used for image acquisition and quantitative analysis. Time-lapse images were recorded for 20 h at the interval of 15 min unless otherwise described. DAPI, FITC, Texas Red, and Cy5 filters were used for detecting different fluorophores used in this study. Differential Interference Contrast (DIC) imaging is always acquired together with fluorescence imaging. Hoechst33342 staining was detected only one time by UV light illumination and DAPI filter together with DIC image at the beginning of the time-lapse imaging. The nuclear images obtained with Hoechst33342 staining were used for counting initial cell numbers, which is used to normalize the result of other fluorescence imaging in each microscopic field. Quantitative analysis was done by Nikon Element analysis, including thresholding, binary layer, and automated measurement module. For live-cell imaging, 35 mm glass-bottom culture dish (part no.: P35G-1.5-20-C), 6-well (part no.: P06G-1.5-20-F, and 12-well (part no.: P12G-1.5-14-F) glass-bottom culture plates were used (MatTek Life Sciences).

### METs Image Analysis

The monochrome fluorescent image sequences of METs releasing cells were obtained from time-lapse microscopy by cropping and exporting to TIFF format image files. The exported files were opened in Fiji ImageJ (22). Each fluorescence image was converted to 8-bit grayscale image and blurred by a Gaussian filter with sigma (radius) parameter 2.0. The image was then further processed by thresholding to produce a binary image. The thresholding value was applied to the image by adjusting the value from the histogram. The image was then converted to a binary image, and measured the area of each binary image by using analyze particle command. A nuclear area analysis for normal THP-1 cells was also done in the same way.

### Immunofluorescence

The immunofluorescence of citrullinated histone H3 (CitH3) and MPO were performed in this study. The cells treated with rmCIRP were fixed with 4% paraformaldehyde for 15 min at room temperature and washed 3 times with PBS. The fixed cells were then blocked with 5% BSA and 100 mM glycine in PBS for 2 h, followed by the incubation of the primary antibody overnight at 4°C. Rabbit anti-citH3 antibody (1:1,000 dilution) and anti-MPO-FITC (1:1,000 dilution) were mixed in the blocking buffer. The cells were then washed 3 times with PBS and incubated with donkey anti-rabbit antibody-Alexa F594 (Thermo Fisher Scientific, A21207) for 2 h. After washing the cells 3 times with PBS, the immunofluorescence sample was prepared by adding prolong gold antifade (cat. no.: P36934, Thermo Fisher Scientific).

### Confocal Microscopy

High-resolution images of METs structure were obtained by Zeiss Axio Observer equipped with LSM880 confocal microscopy system. The z-stack images of 4 neighboring microscopic fields were obtained using Plan-Apochromat 40×/0.95 Korr M27 objective lens and stitched to make a big image that can visualize the whole structure of METs formed among multiple cells. Using EC Plan-Neofluar 10×/0.30 M27 objective lens wide range of microscopic fields was also captured by the same confocal microscope. The signal of each fluorescence channel was averaged four times. The z-stack images were processed to show maximum-intensity projection. The detailed structure of METs formed by rmCIRP was obtained by a Axio Observer.Z1/7 equipped with Zeiss LSM900 confocal microscopy system. The z-stack images of cells were acquired with Plan-Apochromat 63×/1.40 Oil DIC M27 objective lens. SR-4Y fast acquisition mode of Airyscan and 4× averaging were used. The images obtained by confocal microscope were merged and combined by FIJI ImageJ (22).

### Statistical Analysis

Data represented in the figures are expressed as mean ± SEM. To test the significance of multiple comparison, one-way ANOVA with Turkey method was used. A two-tailed Student *t-test* was applied for two-group comparisons. Significance was considered for p ≤0.05 between study groups. Data analyses and the graph preparation were carried out using GraphPad Prism graphing and statistical software (GraphPad Software, San Diego, CA).

## Results

### eCIRP Induces METosis in a Time- and Dose-Dependent Manner

**Figure 1:**
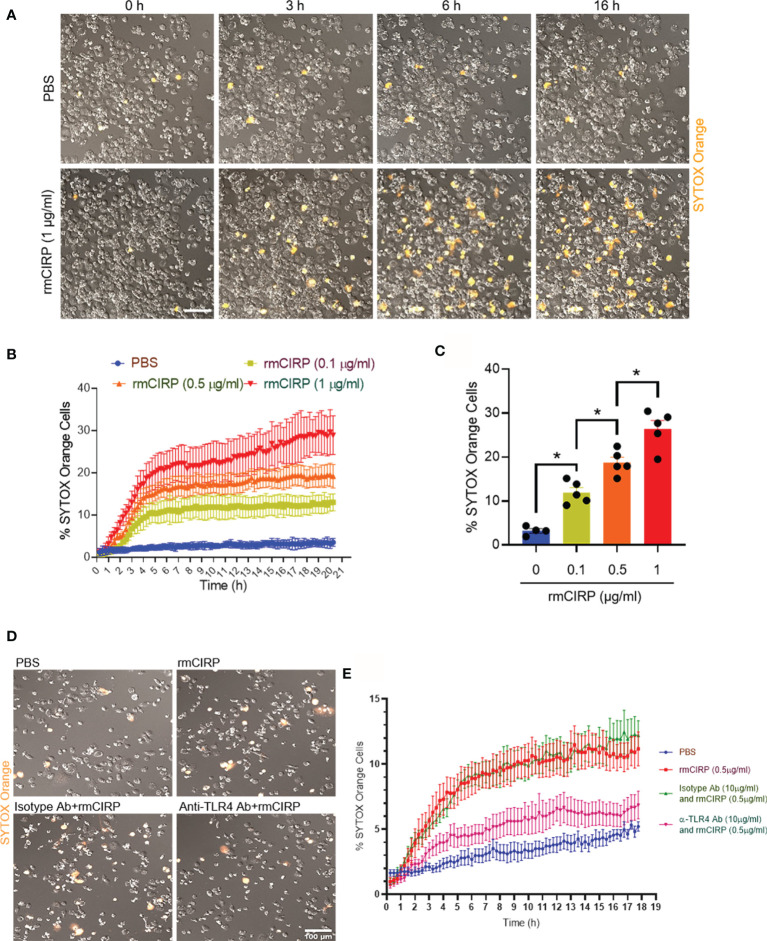
eCIRP induces extracellular trap formation in THP-1 cells. **(A)** THP-1 cells were treated with PBS or rmCIRP (1 µg/ml) in the presence of SYTOX Orange (0.6 µM) and subjected to the time-lapse live-cell imaging immediately after the treatment with rmCIRP. The upper panel shows the cells treated with PBS without rmCIRP, and the lower panel shows the cells treated with rmCIRP at different time points. Scale bar, 100 µm. **(B)** The dose-dependent effect of rmCIRP was quantified by counting the cells positive to SYTOX Orange. **(C)** Data shows the percentage of THP-1 cells positive to SYTOX Orange at 16 h after rmCIRP treatment (n = 4–5 microscopic fields for each condition). One-way ANOVA with Turkey method: *p <0.05. **(D)** THP-1 cells were pre-treated with IgG isotype Abs (10 µg/ml) or anti-TLR4 Abs (10 µg/ml) for 30 min. These cells were then treated with PBS or rmCIRP (0.5 µg/ml) in the presence of SYTOX Orange (0.6 µM) and subjected to the time-lapse live-cell imaging immediately after the treatment with rmCIRP. The time-lapse live-cell imaging data were acquired up to 18 h. The representative microscopic images taken at 18 h of rmCIRP treatment are captured and shown. Scale bar, 100 µm. **(E)** The percentages of SYTOX Orange positive cells in various experimental groups with time are shown.

The treatment of THP-1 cells with a constant dose of rmCIRP increased the frequency of SYTOX Orange positive cells with increasing time. In contrast, the PBS-treated cells did not show SYTOX Orange positivity with time (**Figure 1A** and **Supplementary Video S1**). To determine the kinetics of the SYTOX Orange positivity, we set the time-lapse program. The duration of the imaging was 20 h with 15 min intervals. According to the time-lapse microscopy data, we noticed that the treatment of cells with various doses of rmCIRP at different times increased SYTOX Orange positivity in the cells in a dose- and time-dependent manner (**Figure 1B**). At varied concentrations of rmCIRP, SYTOX Orange positive cells were increased with bi-phasic mode, an initial rapid increase within 4 h, followed by the plateau period with an increased magnitude of METs at an increased concentration of rmCIRP (**Figure 1B**). At 16 h of incubation, the percentages of SYTOX Orange positive cells in the culture with 0, 0.1, 0.5, and 1 µg/ml of rmCIRP were 3.2 ± 0.5, 11.9 ± 1.1, 18.8 ± 1.2, and 26.4 ± 1.9%, respectively (**Figure 1C**). SYTOX Orange is a commonly used reagent to detect extracellular traps (8). TLR4 serves as the putative receptor of eCIRP (1). We found that the THP-1 cells pre-treated with anti-TLR4 Abs significantly decreased rmCIRP-induced MET formation compared to IgG-treated control (**Figures 1D, E**). Therefore, the above finding suggests that eCIRP induces METs through TLR4 pathway.

### Morphological Changes Indicate MET Formation Following eCIRP Stimulation

**Figure 2:**
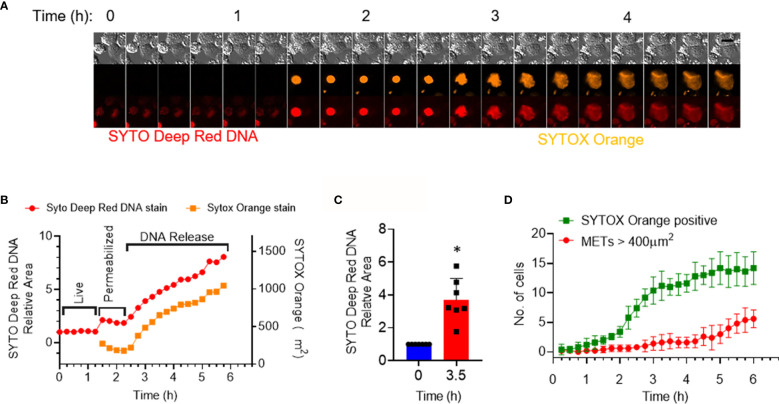
eCIRP stimulation of THP-1 cells causes morphological changes indicating MET formation. THP-1 cells were treated with rmCIRP (1 µg/ml). **(A)** A montage of time-lapse images of a single cell shows the process of MET formation by rmCIRP treatment. Images from the time-lapse microscopy show swelling, permeabilization, and staining of SYTOX Orange in the nucleus and subsequently the release and spread of DNA. SYTO Deep Red DNA staining (bottom panel) shows the nuclear DNA. Scale bar (upper right), 20 µm. **(B)** The nuclear swelling and DNA release were quantified by the area measurement of SYTO deep red DNA and SYTOX Orange staining depicted in panel **(A)**. It shows two stages of morphological changes, permeabilization (nuclear swelling) and subsequent release of DNA. **(C)** The fold change of the area of SYTO Deep Red DNA staining of 7 individual cells. *p <0.05 vs. 0 h. **(D)** The number of total SYTOX Orange positive cells and METs is represented in this graph. METs are bigger than 400 µm^2^ and are plotted through time. The data collected was the average of 5 different microscopic fields from the cell culture treated with 1 µg/ml rmCIRP.

We next investigated the shape changes in the macrophages following treatment with rmCIRP. In the dose–response curve, we identified that the cells rapidly responded to rmCIRP within the first 4 h after its treatment (**Figure 1B**). We, therefore, focused on the events that occurred within 4 h after the rmCIRP treatment. It was noted that the cells underwent a series of morphological changes, which include initial nuclear swelling, followed by mild shrinkage as determined by the area of the SYTO Deep Red DNA and SYTOX Orange staining, and plasma membrane leakage, resulting in the incorporation of SYTOX orange in the cells, followed by the METs release, during treatment with rmCIRP (**Figures 2A, B** and **Supplementary Figure S1A**, **Supplementary Video S2**). At 3.5 h after the rmCIRP treatment, the average SYTO Deep Red positive area of the cells analyzed was 3.7 ± 0.5 folds bigger than their nuclear size initially measured (**Figure 2C**). We further analyzed the number of METs structures in the microscopic field observed based on this analysis. The result showed that 42.8% of cells (6 out of 14 cells) positive to SYTOX Orange represented METs releasing cells at 6 h of rmCIRP treatment (**Figure 2D**). To evaluate the number of METs detected by SYTOX Orange staining in microscopic fields, the cutoff value of METs size was determined by measuring the average size of the nucleus of resting cells. We obtained fluorescence nucleus images of THP-1 cells treated with PBS. There was no METs structure observed from the image of the PBS-treated cells. THP-1 cells were stained with Hoechst 33342, and the fluorescent image was taken for counting cell number (604 cells counted) and area of each nucleus of the cells. The mean area of the nucleus was 150.8 ± 3.0 µm^2^ (**Supplementary Figure S1B**). The fluorescent objects less than 5 µm^2^ were ignored. The resulting area of the nucleus analyzed from a microscopic field showed right-skewed distribution ranging from 5.074 to 467.6 µm^2^. The median of the distribution was 150.4 µm^2^ with 114.2 and 195.6 µm^2^ for 25 and 75 percentiles, respectively (**Supplementary Figure S1B**). Due to the existing bi-nucleated cells in the population, we used 400 µm^2^ (about 2.6-fold larger area than the average area of the normal nucleus) as a cutoff value to count the number of METs formed by the rmCIRP treatment. These data confirm METosis following eCIRP treatment of THP-1 cells based on the distinct morphological features of the treated cells.

### Web-Like Structures are Formed in Macrophages Treated With eCIRP

**Figure 3:**
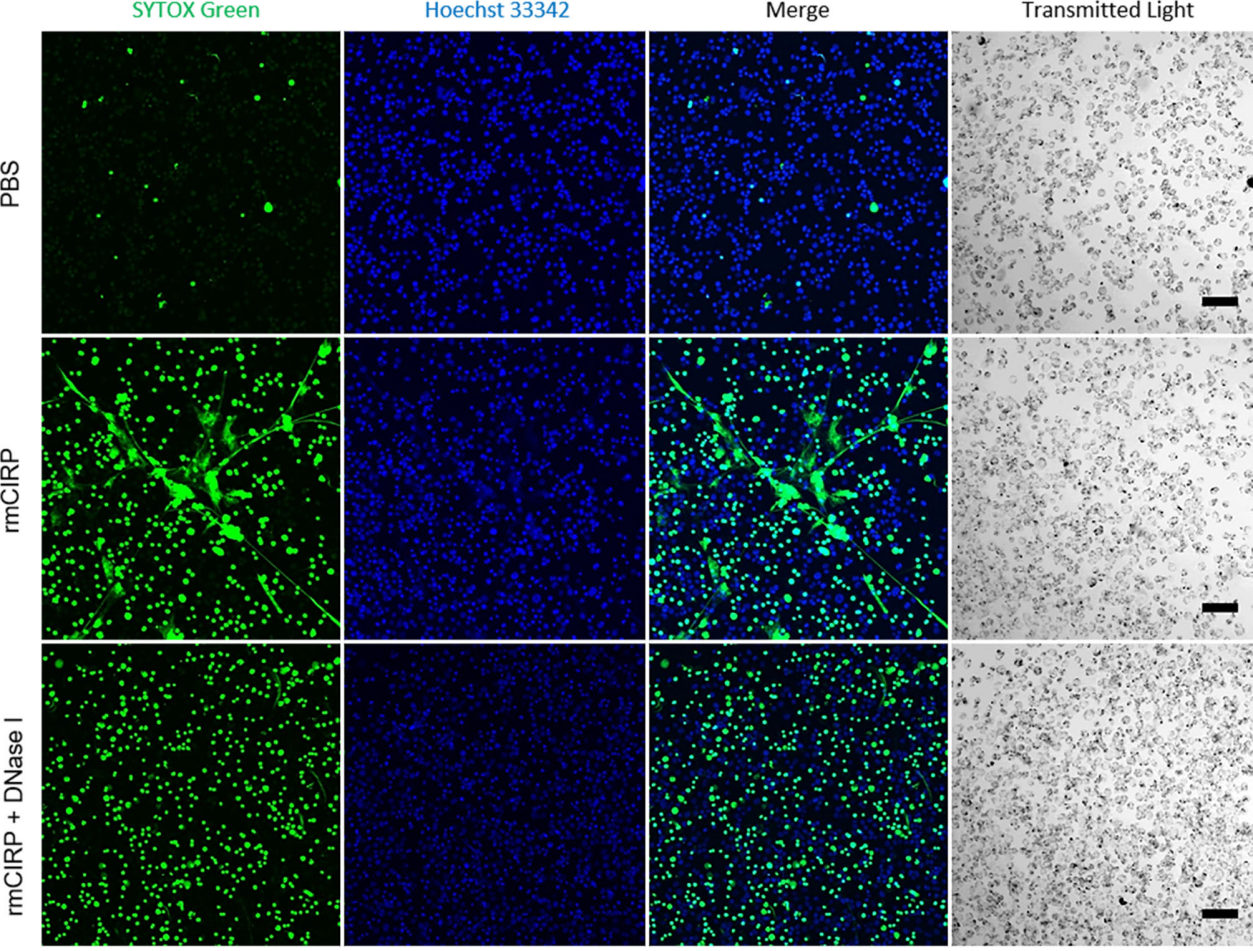
eCIRP-induced METs are eliminated by DNase I treatment. THP-1 cells were treated with PBS (top panel), rmCIRP (1 µg/ml) (middle panel), or rmCIRP (µg/ml) and DNase I together (bottom panel) and incubated for 6 h and followed by the incubation of Hoechst 33342 and SYTOX Green stain for 1 h. The images were taken with a Zeiss LSM880 confocal microscope equipped with EC Plan-Neofluar 10×/0.30 M27 objective lens. While control cells did not show any MET formation by treatment of the buffer solution alone, rmCIRP treatment induced a massive network of extracellular traps in the culture. However, co-treatment of rmCIRP and DNase I (10 U/ml) eliminated the extracellular traps in the culture with a similar number of SYTOX Green positive cells, comparable to the number of rmCIRP alone treated. The acquired z-stack scan images were then processed to make maximum intensity projection by Fiji ImageJ. Scale bar, 100 µm.

The structure of METs was further investigated by observing THP-1 cells under confocal microscopy. METs induced by the rmCIRP treatment formed a widespread inter-connected web-like network (**Figure 3**). Nonetheless, the web-like structure disappeared by the treatment with deoxyribonuclease I (DNase I), an endonuclease that cleaves DNA. At the same time, the intracellular DNA staining by SYTOX Green was still visible, indicating that DNase I hydrolyzed mostly the extracellular DNA. It is to be noted that the inter-connected web-like METs structure observed was labile to sheering forces such as shaking (**Supplementary Video S3**). In the video, the microscope base was tapped gently to shake the cell culture, and the METs were buoyant and loosely attached to the surface of the culture dish. The web-like METs structure was also observed in primary mouse peritoneal macrophages treated with rmCIRP for 20 h (**Supplementary Figure S2**).

### Extracellular DNA Release Accompanies Citrullinated Histone H3 and Myeloperoxidase

**Figure 4:**
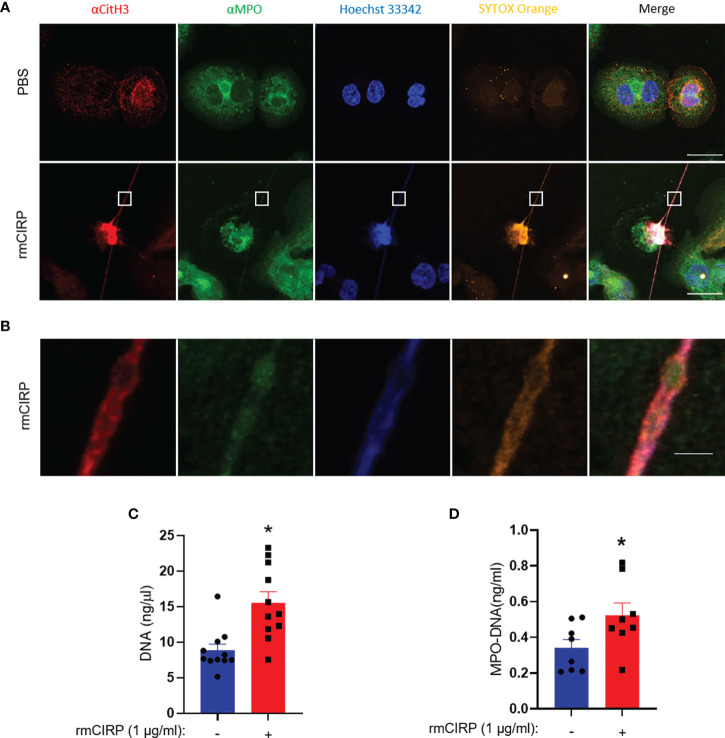
CitH3 and MPO are released with DNA from THP-1 cells treated with rmCIRP. **(A)** rmCIRP (1 µg/ml) induces citrullination of histone H3 in the released DNA. The immunofluorescence microscopy shows that rmCIRP significantly increases the citrullination of histone H3 of nuclear DNA, whereas no citrullinated histone H3 was detected in the nucleus of control cells. Scale bar, 20 µm. **(B)** The blown-up images of the white boxes show that CitH3 and MPO are colocalized in SYTOX Orange positive extracellular DNA. Scale bar, 2 µm. **(C)** METs released in the culture media were quantified by UV spectrometer. Student *t*-test: *p <0.01 vs. PBS (without rmCIRP). **(D)** ELISA quantified MPO–DNA released in the culture media. Student *t*-test: *p <0.05 vs. PBS (without rmCIRP).

The cells were treated with rmCIRP for 6 h and stained with antibodies against CitH3 and MPO. The PBS-treated cells neither showed extracellular DNA nor did they have any CitH3 and MPO staining. Upon treatment of THP-1 cells with rmCIRP, the cells released METs as evidenced by the extracellular DNA, together with citH3 and MPO (**Figure 4A**). The image showed a typical dead cell-like morphology with a deformed nucleus and a long stretch of extracellular DNA stained with SYTOX Orange. The cell showed increased citrullinated histone H3 and MPO staining embedded into the extracellular DNA (**Figure 4A**). The blow-up image of the small rectangular box in the merged image in **Figure 4A** showed details of the extracellular DNA region (**Figure 4B**). We further confirmed the presence of extracellular DNA and its associated MPO quantitatively. We found that the treatment of THP-1 cells with rmCIRP significantly increased the DNA contents by 68% (PBS: 8.8 ± 0.9 ng/µl; rmCIRP: 15.6 ± 1.6 ng/µl) (**Figure 4C**). Similarly, we also found that the treatment of macrophages with rmCIRP significantly increased the MPO levels in the METs by 35% (PBS: 0.34 ± 0.05 ng/ml; rmCIRP: 0.52 ± 0.07 ng/ml) (**Figure 4D**).

### eCIRP Induces MET Formation Through GSDMD Activation

**Figure 5:**
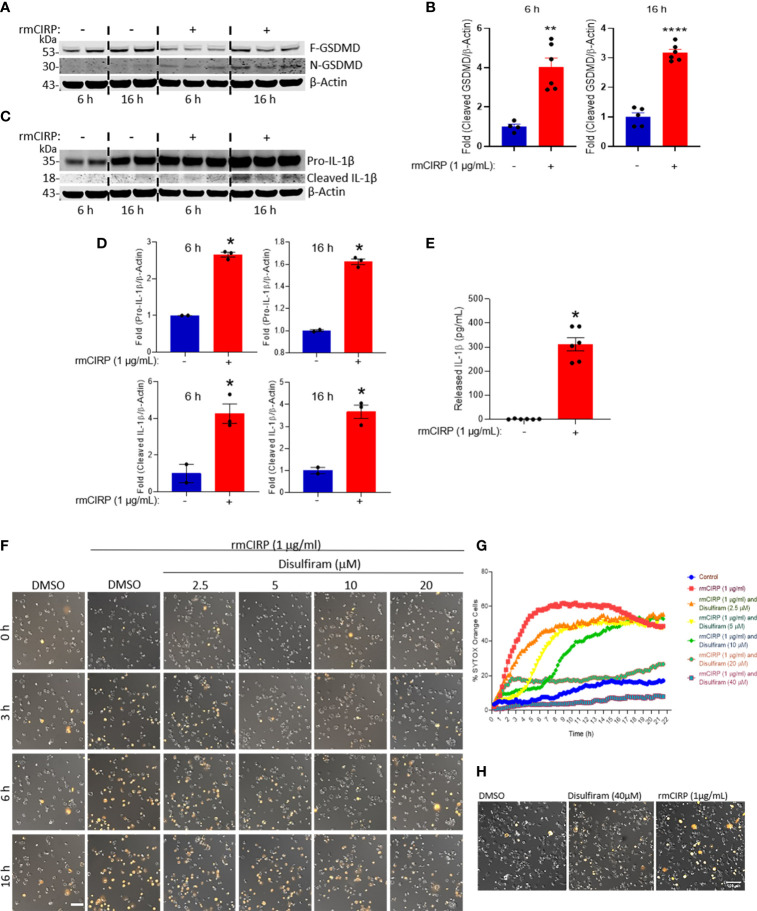
eCIRP induces MET formation through GSDMD activation in THP-1 cells. **(A)** rmCIRP (1 µg/ml) treatment increases the proteolytic cleavage of GSDMD, which indicates the activation of GSDMD. Lysates of THP-1 cells with or without rmCIRP treatment for 6 or 16 h were subjected to the Western blotting with antibody specific to cleaved N-GSDMD fragment, then probed with antibody specific to full-length GSDMD and β-actin after stripping the membrane. Full gel images are shown in **Supplementary Figure S3**. **(B)** The Western blot shows a significant increase of pore-forming N-GSDMD fragments after rmCIRP treatment. The intensity of the bands detected was quantified by densitometry. Student *t*-test: **p <0.01 vs. PBS (without rmCIRP) at 6 h, ****p <0.001 vs. PBS (without rmCIRP) at 16 h. **(C)** Lysates of THP-1 cells with or without rmCIRP treatment for 6 or 16 h were subjected to the Western blotting with antibody specific to human IL-1β, that detect pro- (35 kD) and mature IL-1β (18 kD). Full gel images are shown in **Supplementary Figure S3**. **(D)** The intensity of the bands detected was quantified by densitometry. Student *t*-test: *p <0.05 vs. PBS (without rmCIRP) at 6 h, *p <0.05 vs. PBS (without rmCIRP) at 16 h. **(E)** THP-1 cells were treated with rmCIRP for 16 h and then cell culture supernatants were collected. IL-1β levels in the culture supernatants were detected by ELISA. Student *t*-test: *p <0.05 vs. PBS (without rmCIRP) at 16 h. **(F)** Pretreatment of disulfiram at various doses suppresses the cell permeabilization and the formation of extracellular traps by rmCIRP in THP-1 cells. The inhibition effect of disulfiram is dose-dependently increased. Cells were treated with disulfiram and SYTOX Orange (0.6 µM) for 30 min. THP-1 cells were subjected to the time-lapse live cell imaging immediately after the treatment of rmCIRP (1 µg/ml). Scale bar, 100 µm. **(G)** The inhibitory effect of disulfiram on eCIRP-induced MET formation is dose-dependently increased. The inhibitor disulfiram was pretreated 30 min before rmCIRP (1 µg/ml), and the cells were subjected to the time-lapse live-cell imaging immediately after the treatment of rmCIRP. The effect of disulfiram was quantified by counting the cells positive to SYTOX Orange. **(H)** Cell viability assay after disulfiram treatment using SYTOX Orange. THP-1 cells were treated with DMSO, Disulfiram only at a concentration of 40 µM, and rmCIRP (1 µg/ml), separately. Cells were simultaneously added with 0.6 µM of SYTOX Orange were subjected to the time-lapse live-cell imaging. After 16 h, microscopic images were acquired. Representative images are shown. Scale bar, 100 µm.

Previously, the extracellular trap formation in neutrophils was shown to be mediated through the activation of GSDMD (11), which is reflected by the cleavage of full-length GSDMD into N- and C-terminal cleaved GSDMD (10). The N-terminal GSDMD causes pore formation in the cell membrane through which the extracellular DNA is expelled out of the cells. Here, we found that the treatment of macrophages with rmCIRP significantly increased the cleaved (N-terminal) form of GSDMD by 4- and 3-fold at 6 and 16 h incubation, respectively (**Figures 5A, B** and **Supplementary Figure S3**). Since caspase-1 activation leads to the cleavage of pro-IL-1β to mature IL-1β and its release to the extracellular spaces through GSDMD pores of pyroptotic cells (10), we assessed pro-IL-1β and cleaved (mature) form of IL-1β in the THP-1 cell lysates and IL-1β levels in the culture supernatants of rmCIRP-treated THP-1 cells. We found that the treatment of THP-1 cells with rmCIRP at 6 and 16 h significantly increased the expression of full-length (pro-) IL-1β compared to their respective PBS-treated control cells **(** **Figures 5C, D** and **Supplementary Figure S3** **)**. Besides the expression of full-length IL-1β, we also determined the cleaved form of IL-1β in rmCIRP-treated THP-1 cells. However, we noticed that even though the expression of pro-IL-1β was predominantly observed, the presence of the cleaved IL-1β as a light band intensity could be because as soon as the mature cleaved form of IL-1β was produced, it was immediately released from the cells, giving rise to faint bands in the total cell lysates **(** **Figures 5C, D** and **Supplementary Figure S3** **)**. Furthermore, we assessed the IL-1β levels in the culture medium, which revealed that IL-1β in the culture supernatants were significantly higher in rmCIRP-treated THP-1 cells compared to the PBS-treated cells **(** **Figure 5E** **)**. To determine whether GSDMD activation causes MET formation by the THP-1 cells treated with rmCIRP, we treated cells with an antagonist of GSDMD activation, disulfiram, which interferes with the formation of N-GSDMD multimerization (23). We tested disulfiram together with rmCIRP to THP-1 cells and compared it to rmCIRP alone. The effect of disulfiram was determined by time-lapse live-cell imaging with SYTOX Orange staining to measure MET formation. The result was obtained by taking images of DIC and fluorescence images of SYTOX Orange staining (**Figure 5F**). The representative images from the experiment clearly showed a decreased number of SYTOX Orange positive cells with disulfiram treatment (**Figure 5F**). Cell permeabilization was inhibited initially but eventually happened later in the cells treated with disulfiram at low concentrations. As disulfiram covalently modifies GSDMD and prevents it from forming membrane pores (23), we speculated that the disulfiram gradually depleted through time and failed to prevent the newly expressed GSDMD from pore formation. It is noted that increasing the concentration of disulfiram delays the onset of cell permeabilization. The test showed a dose-dependent suppression of SYTOX Orange permeabilization by rmCIRP through time (**Figure 5G**). In addition, we tested the cellular viability after disulfiram treatment. We found that the treatment of the cells with only disulfiram at a dose of 40 µM, the highest dose of it used to inhibit eCIRP-induced GSDMD activation and MET formation **(** **Figures 5F, G** **)**, showed no adverse effects on the cell viability as the results were compared with the DMSO-treated control **(** **Figure 5H** **)**. These results suggest that eCIRP induces MET formation in THP-1 cells through GSDMD activation.

### Inhibition of Caspase-1 Activation Reduces eCIRP-Induced METosis

**Figure 6:**
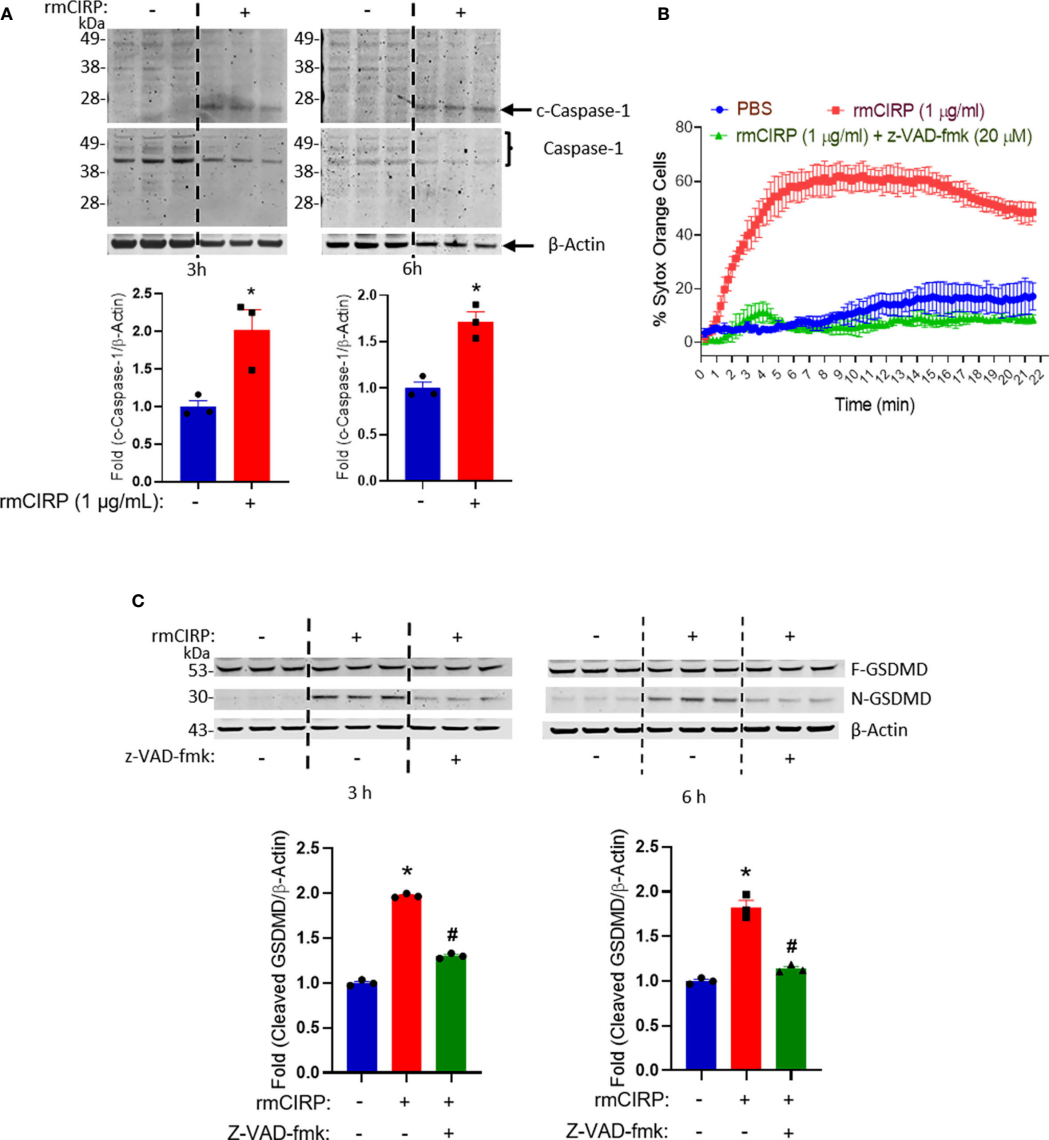
Caspase-1 regulates eCIRP-induced GSDMD-mediated MET formation in THP-1 cells. **(A)** Caspase-1 is cleaved by the treatment of rmCIRP (1 µg/ml) in THP-1 cells. The cleavage of caspase-1 was observed at 3 and 6 h after the treatment of rmCIRP (1 µg/ml). The Western blot of cleaved caspase-1 was detected first. Full-length GSDMD and β-actin were probed at the same time after stripping the anti-cleaved GSDMD antibody. The intensity of bands detected was quantified by densitometry. Student *t*-test: *p <0.05 vs. PBS (without rmCIRP) at 3 h, *p <0.01 vs. PBS (without rmCIRP) at 6 h. **(B)** The effect of rmCIRP (1 µg/ml) in THP-1 cells is significantly inhibited by z-VAD-fmk (20 µM), a pan-caspase inhibitor. The inhibitor was pre-treated 30 min before rmCIRP (1 µg/ml) treatment, and the cells were subjected to the time-lapse live-cell imaging immediately after the treatment of rmCIRP. The effect of z-VAD-fmk was quantified by counting the cells positive to SYTOX Orange. **(C)** Treatment of z-VAD-fmk at a dose of 20 µM inhibits the activation of GSDMD by rmCIRP (1 µg/ml). The western blot of cleaved GSDMD was done first. Full length GSDMD and β-actin were probed at the same time after stripping off the anti-cleaved GSDMD antibody. The band intensity was determined by densitometry and compared to the control group. Full gel images are shown in **Supplementary Figure S4**. One-way ANOVA with Turkey method: *p < 0.01 vs. PBS; ^#^p < 0.01 vs. mCIRP for 3 or 6 h treatment study.

To determine the upstream signaling pathway of the activation of GSDMD induced by rmCIRP we investigated whether caspase-1 is the regulator of GSDMD activation by rmCIRP. The treatment of THP-1 cells with rmCIRP significantly increased the activation of the caspase-1 as revealed by the increased levels of the cleaved form of caspase-1 as early as 3 h by 2-fold and at a later time point, 6 h by 1.7-fold of the treatment of rmCIRP compared to the PBS-treated control (**Figure 6A**). We next determined the effect of caspase-1 inhibition by z-VAD-fmk, a pan-caspase inhibitor, on eCIRP-induced MET formation. We found that the treatment of z-VAD-fmk significantly inhibited the formation of METs by the THP-1 cells treated with rmCIRP in a time-dependent manner (**Figure 6B**). Even though there was a slight increase in the SYTOX Orange staining around 4 h of z-VAD-fmk treatment (11.2 ± 1.8%), it was significantly lower than that of rmCIRP alone (48.5 ± 2.3%). The initial slightly increased staining of SYTOX Orange in the z-VAD group could be due to the acute effect of rmCIRP that opposed the inhibitory effect of z-VAD-fmk and resulted in cell death. The decrease in the number of SYTOX Orange staining after 4 h time point in z-VAD-fmk treated cells was because the dead cells escaped from the microscopic field by detaching from the surface of the culture dish. To confirm that GSDMD activation was regulated by caspase-1, we found that the treatment of z-VAD-fmk showed a significant decrease in N-GSDMD levels as induced by rmCIRP. N-GSDMD was increased by about 1.9- and 1.8-fold for 3 and 6 h incubation with rmCIRP, respectively. By treating z-VAD-fmk, eCIRP-mediated GSDMD activation was significantly decreased at 3 and 6 h incubation of rmCIRP (**Figure 6C** and **Supplementary Figure S4**). Collectively, these data suggest that eCIRP-induces METosis through caspase-1-dependent GSDMD pore formation.

## Discussion

In this study, we have convincingly identified eCIRP as an inducer of MET formation in human monocytes, THP-1 cells, and in murine peritoneal macrophages. We confirmed eCIRP-induced MET formation using several tools, namely, time-lapse fluorescence microscopy (video imaging), colorimetry, and ELISA. We also confirmed METs by their unique morphology of increased nuclear size (400 µm^2^, about 2.6-fold larger area than the average area of the normal nucleus as a cutoff value to determine the MET positive cells). THP-1 cells are widely used for studying the innate-immune function of macrophages (14). Here, we revealed that eCIRP induced MET formation *via* GSDMD activation, which was confirmed by the finding that blocking GSDMD with its pharmacologic inhibitor, disulfiram, decreased MET formation. Since GSDMD activation occurs through the activation of the upstream molecule, caspase-1 activation (10, 24, 25), we identified that blocking caspase-1 using a pan-caspase inhibitor, z-VAD-fmk (25) abrogated GSDMD activation and MET formation induced by eCIRP in macrophages. Furthermore, we previously determined that eCIRP induces NLRP3 inflammasome and promotes pyroptosis, an inflammatory cell death process, *via* caspase-1 activation (17). In light of our previous findings on eCIRP–inflammasome–pyroptosis (17), it is conceivable that eCIRP-mediated GSDMD activation and MET formation occurs due to inflammasome activation.

eCIRP was shown to activate the macrophages to release cytokines and chemokines (2). The identification of eCIRP’s new role in MET formation reflected its broader functions to induce innate immunity. Even though many papers have demonstrated extracellular trap formation in macrophages by various stimuli (i.e., bacteria, virus, LPS, chemokines, and PMA) (13, 14), here we identified eCIRP as a new stimulant of METosis. To firmly determine eCIRP’s effects on METs we employed various tools such as confocal microscopy, time-lapse live microscopic imaging, colorimetry, and ELISA to detect METs in eCIRP-treated macrophages. While detecting METs using the impermeant membrane dyes SYTOX Orange and SYTOX Green, we noticed that following rmCIRP stimulation, a significant portion of the cells became stained with these dyes throughout the cells, making it difficult to distinguish them from inside vs. outside staining. Since these dyes have also been used to assess dead cells, we cannot rule out the possibility of dead cell staining. Like other cell death processes, such as apoptosis, necrosis, and pyroptosis, METosis is also a cell death process where following extracellular trap formation, cells irreversibly undergo dying. This raises the possibility that METotic cells die, leading to incorporating these dyes and staining the entire cells instead of only the extracellular parts. Several types of extracellular traps in neutrophils have been reported, namely, vital, suicidal, and mitochondrial (7, 8). Considering this fact, future studies to differentiate vital, suicidal, or mitochondrial METs will be of interest. Nevertheless, using the time lapse live imaging tool, we found the cell swelling and extrusion of chromatin contents following eCIRP stimulation indicating valid METosis. In addition, using the other markers like MPO, citH3 Abs, we proved METosis following eCIRP stimulation, as expulsion of these molecules within the extracellular DNA is unique for METosis, and thus can distinguish it from other types of cells death.

We adopted a novel approach of MET detection by assessing the size of the nuclear contents by DNA staining dyes and visualizing them in a time-lapse microscope. In oppose to apoptosis, where shrinkage of cellular contents usually happens (26), we found initial swelling of the nucleus, indicating dispersion and expulsion of nuclear contents suggesting METosis. Citrullination of histone H3 is the hallmark of extracellular trap formation (8). Peptidyl arginine deaminase 4 (PAD4) plays a critical role in histone H3 citrullination (27). Therefore, apart from studying GSDMD-mediated MET formation, we also assessed PAD4’s involvement in eCIRP-mediated METosis. We found that treatment of the macrophages with PAD4 inhibitor dramatically decreased eCIRP-mediated METosis, indicating that citrullination of histone H3 for METosis may also be required **(** **Supplementary Figure S5** **)**. Since our previous study on eCIRP stimulated NET formation demonstrated that eCIRP induces PAD4 expression and enzymatic activation (9), we speculate that macrophages treated with rmCIRP can also induce PAD4 activation for MET formation.

We previously reported that eCIRP induces neutrophils to form extracellular traps in sepsis, containing citrullinated histone H3, MPO, and extracellular DNA, activating the immune system to promote inflammation (9, 20, 28). Given that eCIRP-induced METs have the same molecules as NETs, it is expected that METs can induce inflammation and exhibit harmful effects in various inflammatory diseases. In line with our findings, previous studies reported that during sepsis and inflammatory diseases, METs were formed, causing detrimental effects (13, 14, 29). Since both eCIRP and METs are elevated in sepsis, there might have a relationship between these two molecules in sepsis. Further studies determining the *in vivo* effects of eCIRP in MET formation or the inhibition of eCIRP in sepsis mice to decrease the levels of METosis would be of great potential. Our previous study showed that neutrophils treated with eCIRP generate a novel neutrophil subtype, promoting increased NET formation (28). Akin to that study, identifying novel surface markers or the polarization of macrophages following eCIRP treatment to stimulate MET-forming macrophages will be interesting.

In this study, we treated THP-1 cells with z-VAD-fmk, and then after stimulating these cells with rmCIRP, we determined METs. z-VAD-fmk is a pan-caspase inhibitor that irreversibly binds to the catalytic site of the caspases (30). It inhibits murine caspases, i.e., caspase-1, -3, and -11, the ortholog of human caspase-4 and -5 (31–33). Upon activation, caspase-1 processes IL-1β and IL-18, and GSDMD, promoting inflammation and pyroptosis (32). Indeed, z-VAD-fmk is widely used to investigate inflammasome activation. By contrast, it is also used as an apoptosis inhibitor, as in THP-1 cells z-VAD-fmk was shown to inhibit caspase-3-dependent apoptosis (34). Through its inhibitory activity, z-VAD-fmk can reduce inflammation and pyroptosis, blocks the induction of caspase-3-mediated apoptosis, and trigger necroptosis (31–33). With these functions of z-VAD-fmk, it is obvious that z-VAD-fmk inhibited eCIRP-induced pyroptosis and possibly apoptosis. While eCIRP is a ligand for TLR4 receptor, which strongly indicates pyroptosis, several studies have shown that apoptosis/caspase-3 might also be activated upon classical pyroptotic stimulus such as LPS/nigericin (35). To distinguish the effect of eCIRP for bidirectional crosstalk between pyroptosis and apoptosis, further studies with macrophages treated with anti-caspase-3 and anti-PARP antibodies to see eCIRP’s effects on pyroptosis and METosis will be of interest. In the current study, we studied eCIRP’s effects on MET formation in human monocyte/macrophage cell line, THP-1 cells. However, the inclusion of human peripheral blood mononuclear cells from healthy subjects or patients to study eCIRP’s effects on METs may further strengthen our findings and establish clinical relevancy. Mouse and human CIRP proteins’ amino acids sequence show >95% sequence homology between them (36). We prepared rmCIRP in-house and validated its purity and functions (1). We did not prepare recombinant human (rh) CIRP because mouse and human CIRPs are almost similar. We, therefore, used rmCIRP in our study.

In conclusion, while GSDMD activation to form extracellular traps in neutrophils has been reported before (11), in the context of macrophages, both eCIRP-induced METosis, and caspase-1-mediated GSDMD activation to form METs were not studied. Thus, our findings will shed light on the pathways of innate immune function of macrophages, encompassing the novel pathophysiology of inflammatory diseases.

## Data Availability Statement

The original contributions presented in the study are included in the article/**Supplementary Material**. Further inquiries can be directed to the corresponding authors.

## Ethics Statement

This study was approved by the Institutional Animal Care and Use Committee of the Feinstein Institutes for Medical Research.

## Author Contributions

YL and MA outlined the experimental design. YL performed the METs assays. BR and CT performed caspase-1 and GSDMD assays. YL, MA, and PW analyzed the data. YL and MA prepared the figures. MA and YL wrote the manuscript. PW reviewed and edited the manuscript. PW conceived the idea. MA and PW supervised the project. All authors contributed to the article and approved the submitted version.

## Funding

This study was supported by the National Institutes of Health (NIH) grants R35GM118337 and U01AI133655 (PW), and R01GM129633 (MA).

## Conflict of Interest

The authors declare that the research was conducted in the absence of any commercial or financial relationships that could be construed as a potential conflict of interest.

## Publisher’s Note

All claims expressed in this article are solely those of the authors and do not necessarily represent those of their affiliated organizations, or those of the publisher, the editors and the reviewers. Any product that may be evaluated in this article, or claim that may be made by its manufacturer, is not guaranteed or endorsed by the publisher.

## Supplementary Material

Supplementary Figure S1 rmCIRP induces extracellular trap formation in THP-1 cells. **(A)** Montage of time-lapse images of two single cells show the process of MET formation by rmCIRP treatment. Images from the time-lapse microscopy shows swelling, permeabilization, and staining of SYTOX Orange in the nucleus and subsequently the release and spread of DNA. SYTO Deep Red DNA staining (bottom panel) shows the nuclear DNA. Scale bar (upper right), 20 µm. Nuclear area was measured with ImageJ. **(B)** To evaluate the number of METs detected by SYTOX Orange staining in microscopic fields, the cutoff value of METs size was determined by measuring the average size of the nucleus of resting cells. We obtained fluorescence nucleus images of THP-1 cells treated with PBS. There was no METs structure observed from the image of the PBS-treated cells. THP-1 cells were stained with Hoechst 33342, and the fluorescent image was taken for counting cell number (604 cells counted) and area of each nucleus of the cells. The mean area of the nucleus was 150.8 ± 3.0 µm^2^. The fluorescent objects less than 5 µm^2^ were ignored. The resulting area of the nucleus analyzed from a microscopic field showed right-skewed distribution ranging from 5.074 to 467.6 µm^2^. The median of the distribution was 150.4 µm^2^ with 114.2 µm^2^ and 195.6 µm^2^ for 25 and 75 percentiles, respectively.

Supplementary Figure S2 eCIRP induces MET formation in murine peritoneal macrophages. DNase I treatment significantly decreased the SYTOX Green positive cells. DNase I (10 U/mL) was added to the cell culture with rmCIRP at the same. The effect of DNase I was quantified by counting the cells positive to SYTOX Green. Peritoneal macrophages incubated with rmCIRP (1 µg/mL) for 20 h *in vitro* shows significant release of extracellular traps, which become disappeared by the treatment of DNase I (10 U/mL). The images were taken by confocal microscopy after staining the cells with SYTOX Green and Hoechst 33342 for 1 h.

Supplementary Figure S3 Full gel image of **Figures 5A, C**.

Supplementary Figure S4 Full gel image of **Figure 6C**.

Supplementary Figure S5 METs released by rmCIRP is significantly inhibited by PAD4 inhibition. Inhibition of the activity of peptidylarginine deiminase 4 (PAD4) significantly decreased the MET formation. Cl-amidine was added to the cell culture 30 min prior to rmCIRP treatment. The effect of Cl-amidine was quantified by counting the cells positive to SYTOX Orange.

Supplementary Video S1 Time-lapse live video of THP-1 cells treated with or without rmCIRP. The video was captured by Nikon Element software. The video was made by merging DIC (Differential Interference Contrast) and SYTOX Orange images of each time point acquired. The video is for 20 h time-lapse imaging and the interval is 15 min. Images in **Figure 1A** were selected from the same time-lapse data generated from this video. Scale bar, 100 µm.

Supplementary Video S2 Time-lapse live video of THP-1 cells forming METs. The video was captured by Nikon Element software. It was made by combining DIC, SYTOX Orange, and SYTO Deep Red DNA stain image sequences. The video is for 20 h time-lapse imaging, and the interval is 15 min. **Figure 2A** was made from this video by ImageJ. Scale bar, 10 µm.

Supplementary Video S3 METs released from THP-1 cells by rmCIRP have inter-connected structure, which is vulnerable to sheering forces. The short video was captured by Zeiss confocal LSM880. This video is generated from the data that is used for making the panel of rmCIRP treated condition in **Figure 3**. While taking the video, the microscope was shaken gently by tapping its body. The video was acquired every 3 seconds for 120 seconds (about 2 min). The images of SYTOX Green and Heochst 33342 stain were merged.

## Abbreviations

eCIRP, extracellular cold-inducible RNA-binding protein; METs, macrophage extracellular traps; GSDMD, gasdermin D; PMA, phorbol 12-myristate 13-acetate; TLR4, Toll-like receptor 4; TREM-1, triggering receptor expressed on myeloid cells-1; NETs, neutrophil extracellular traps; MPO, myeloperoxidase; PAD4, peptidyl arginine deiminase 4; DAMP, damage-associated molecular pattern.
