# Supplementary figures and images for: Extracellular CIRP Induces Macrophage Extracellular Trap Formation Via Gasdermin D Activation
Source: Front Immunol. 2021 Dec 23;12:780210. doi: 10.3389/fimmu.2021.780210 (PMC8732379; doi:10.3389/fimmu.2021.780210)

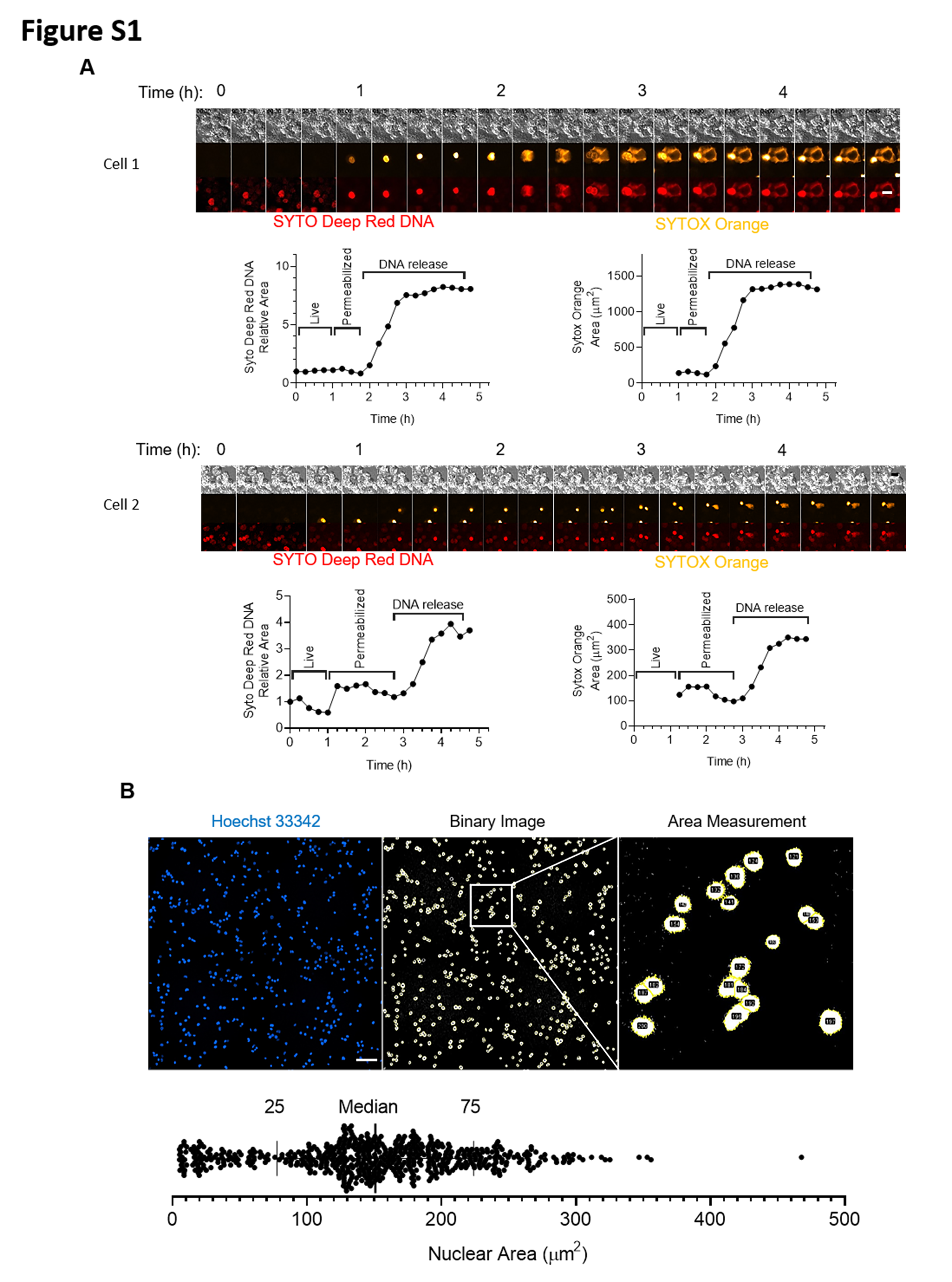

Supplement: Supplementary Figure S1 — rmCIRP induces extracellular trap formation in THP-1 cells. (A) Montage of time-lapse images of two single cells show the process of MET formation by rmCIRP treatment. Images from the time-lapse microscopy shows swelling, permeabilization, and staining of SYTOX Orange in the nucleus and subsequently the release and spread of DNA. SYTO Deep Red DNA staining (bottom panel) shows the nuclear DNA. Scale bar (upper right), 20 µm. Nuclear area was measured with ImageJ. (B) To evaluate the number of METs detected by SYTOX Orange staining in microscopic fields, the cutoff value of METs size was determined by measuring the average size of the nucleus of resting cells. We obtained fluorescence nucleus images of THP-1 cells treated with PBS. There was no METs structure observed from the image of the PBS-treated cells. THP-1 cells were stained with Hoechst 33342, and the fluorescent image was taken for counting cell number (604 cells counted) and area of each nucleus of the cells. The mean area of the nucleus was 150.8 ± 3.0 µm2. The fluorescent objects less than 5 µm2 were ignored. The resulting area of the nucleus analyzed from a microscopic field showed right-skewed distribution ranging from 5.074 to 467.6 µm2. The median of the distribution was 150.4 µm2 with 114.2 µm2 and 195.6 µm2 for 25 and 75 percentiles, respectively. [file Image_1.tif]

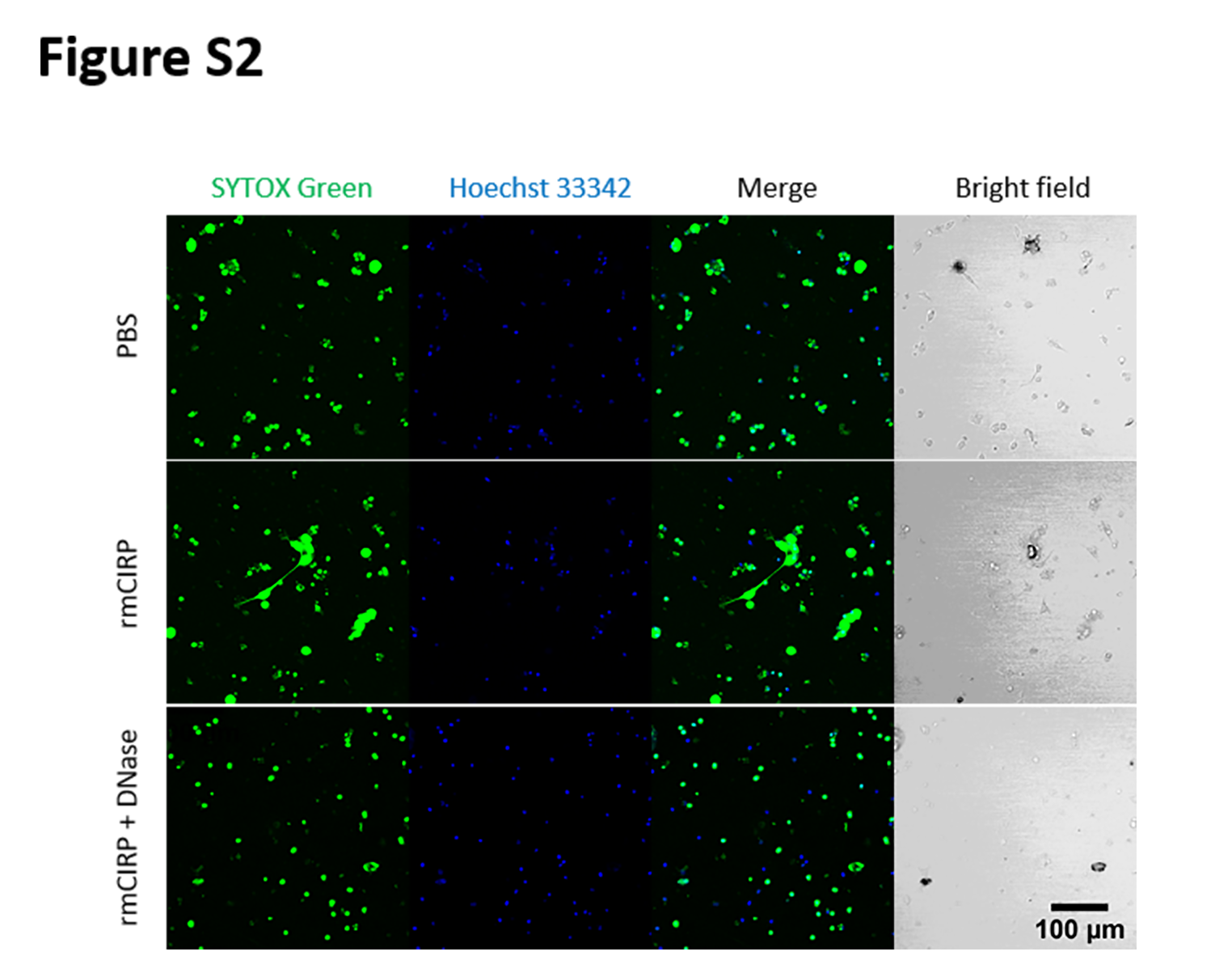

Supplement: Supplementary Figure S2 — eCIRP induces MET formation in murine peritoneal macrophages. DNase I treatment significantly decreased the SYTOX Green positive cells. DNase I (10 U/mL) was added to the cell culture with rmCIRP at the same. The effect of DNase I was quantified by counting the cells positive to SYTOX Green. Peritoneal macrophages incubated with rmCIRP (1 µg/mL) for 20 h in vitro shows significant release of extracellular traps, which become disappeared by the treatment of DNase I (10 U/mL). The images were taken by confocal microscopy after staining the cells with SYTOX Green and Hoechst 33342 for 1 h. [file Image_2.tif]

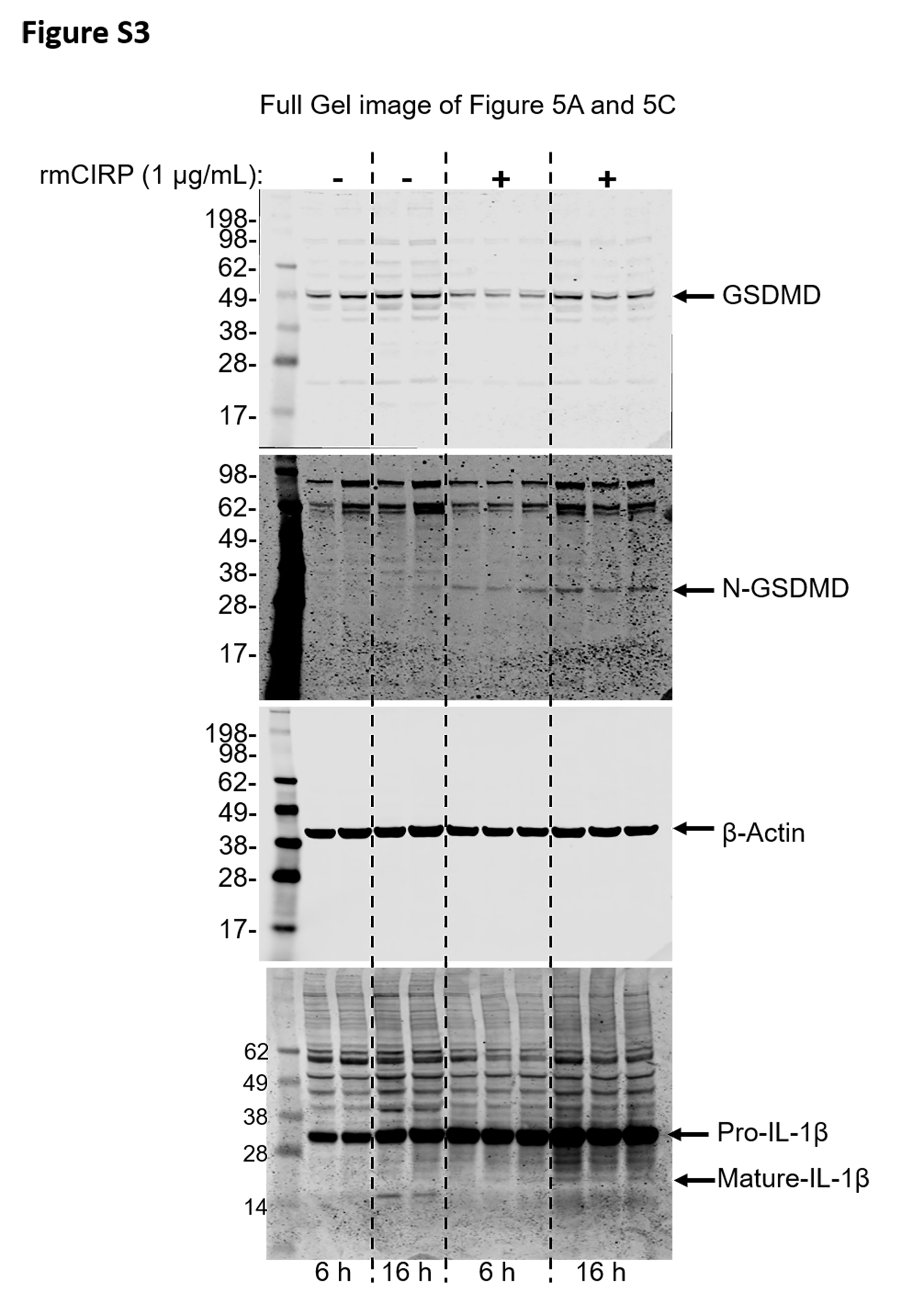

Supplement: Supplementary Figure S3 — Full gel image of Figures 5A, C . [file Image_3.tif]

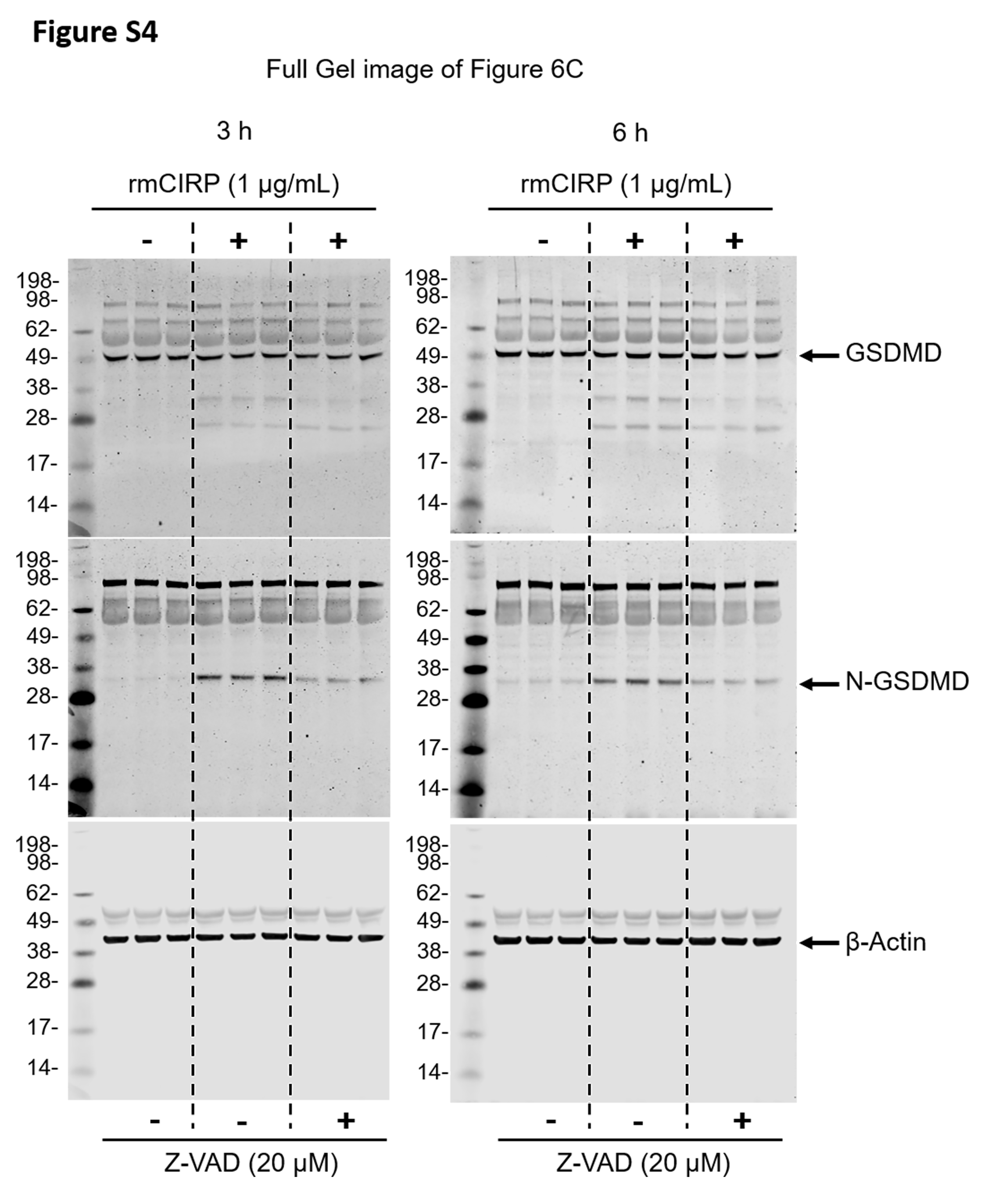

Supplement: Supplementary Figure S4 — Full gel image of Figure 6C . [file Image_4.tif]

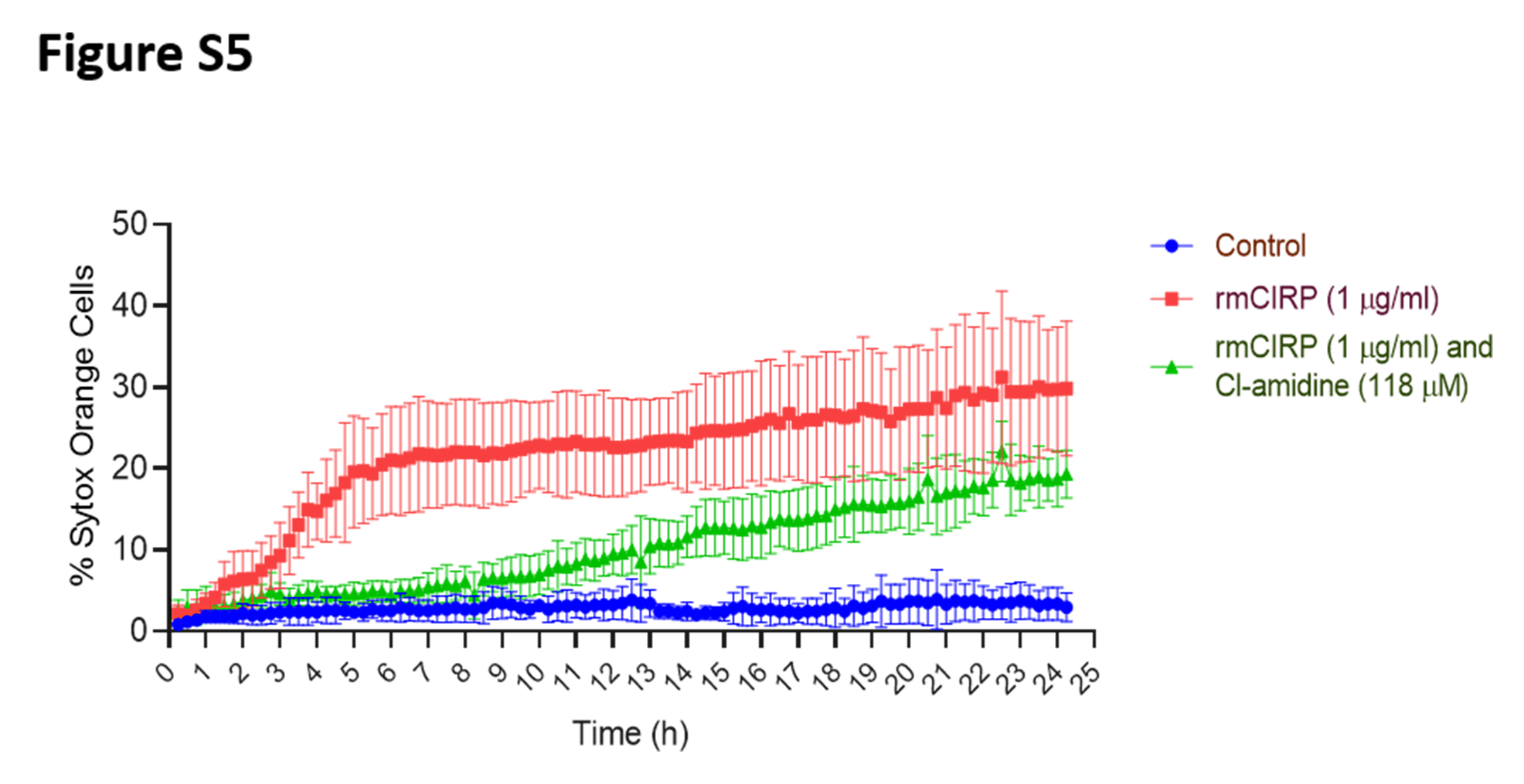

Supplement: Supplementary Figure S5 — METs released by rmCIRP is significantly inhibited by PAD4 inhibition. Inhibition of the activity of peptidylarginine deiminase 4 (PAD4) significantly decreased the MET formation. Cl-amidine was added to the cell culture 30 min prior to rmCIRP treatment. The effect of Cl-amidine was quantified by counting the cells positive to SYTOX Orange. [file Image_5.tif]
